# The Role of Hemoglobin Oxidation Products in Triggering Inflammatory Response Upon Intraventricular Hemorrhage in Premature Infants

**DOI:** 10.3389/fimmu.2020.00228

**Published:** 2020-03-06

**Authors:** Judit Erdei, Andrea Tóth, Andrea Nagy, Benard Bogonko Nyakundi, Zsolt Fejes, Béla Nagy Jr., László Novák, László Bognár, Enikö Balogh, György Paragh, János Kappelmayer, Attila Bácsi, Viktória Jeney

**Affiliations:** ^1^MTA-DE Lendület Vascular Pathophysiology Research Group, Research Centre for Molecular Medicine, Faculty of Medicine, University of Debrecen, Debrecen, Hungary; ^2^Doctoral School of Molecular Cell and Immune Biology, Faculty of Medicine, University of Debrecen, Debrecen, Hungary; ^3^Department of Pediatrics, Faculty of Medicine, University of Debrecen, Debrecen, Hungary; ^4^Department of Laboratory Medicine, Faculty of Medicine, University of Debrecen, Debrecen, Hungary; ^5^Department of Neurosurgery, Faculty of Medicine, University of Debrecen, Debrecen, Hungary; ^6^Department of Internal Medicine, Faculty of Medicine, University of Debrecen, Debrecen, Hungary; ^7^Department of Immunology, Faculty of Medicine, University of Debrecen, Debrecen, Hungary

**Keywords:** intraventricular hemorrhage, hemoglobin, heme, premature infants, cerebrospinal fluid, adhesion molecules, brain endothelial cell, inflammation

## Abstract

Intraventricular hemorrhage (IVH) is a frequent complication of prematurity that is associated with high neonatal mortality and morbidity. IVH is accompanied by red blood cell (RBC) lysis, hemoglobin (Hb) oxidation, and sterile inflammation. Here we investigated whether extracellular Hb, metHb, ferrylHb, and heme contribute to the inflammatory response after IVH. We collected cerebrospinal fluid (CSF) (*n* = 20) from premature infants with grade III IVH at different time points after the onset of IVH. Levels of Hb, metHb, total heme, and free heme were the highest in CSF samples obtained between days 0 and 20 after the onset of IVH and were mostly non-detectable in CSF collected between days 41 and 60 of post-IVH. Besides Hb monomers, we detected cross-linked Hb dimers and tetramers in post-IVH CSF samples obtained in days 0–20 and 21–40, but only Hb tetramers were present in CSF samples obtained after 41–60 days. Vascular cell adhesion molecule-1 (VCAM-1) and interleukin-8 (IL-8) levels were higher in CSF samples obtained between days 0 and 20 than in CSF collected between days 41 and 60 of post-IVH. Concentrations of VCAM-1, intercellular adhesion molecule-1 (ICAM-1), and IL-8 strongly correlated with total heme levels in CSF. Applying the identified heme sources on human brain microvascular endothelial cells revealed that Hb oxidation products and free heme contribute to the inflammatory response. We concluded that RBC lysis, Hb oxidation, and heme release are important components of the inflammatory response in IVH. Pharmacological interventions targeting cell-free Hb, Hb oxidation products, and free heme could have potential to limit the neuroinflammatory response following IVH.

## Introduction

Intraventricular hemorrhage (IVH) is a frequent complication of prematurity, occurring in about 15% to 20% of very low birth-weight (<1,500 g) preterm infants, and its incidence is even higher (∼45%) in extremely low birth-weight infants (500–750 g) (1–3). IVH is associated with high neonatal mortality (20–50%) and increases the risk of neurodevelopmental impairment in the surviving infants beyond the risk associated with prematurity alone (4).

During fetal brain development, neurons and glial cells migrate out from the germinal matrix (GM), a highly cellular and vascularized layer of the brain. The GM is most active between 8 and 28 gestational weeks, and generally absent in term infants (5). In preterm infants, IVH results from bleeding of the GM, because its capillary network is extremely fragile, and unable to regulate cerebral blood flow (6).

IVH in preterm infants leads to systemic inflammation, characterized by elevation of pro-inflammatory cytokines, e.g., tumor necrosis factor alpha (TNF-α), interleukin-8 (IL-8), IL-1β, chemokines such as monocyte adhesion molecule-1, and increased levels of adhesion molecules, i.e., vascular cell adhesion molecule-1 (VCAM-1) and intercellular adhesion molecule-1 (ICAM-1) (7). As a sign of local inflammatory response, the levels of E-selectin, VCAM-1, ICAM-1, and L-selectin were found to be elevated in the cerebrospinal fluid (CSF) of patients after subarachnoid hemorrhage (8).

Rupture of the microvasculature of the GM causes extravasation of red blood cells (RBCs) in the CSF followed by lysis of RBCs. While hemoglobin (Hb) is compartmentalized in RBCs, its oxidation is prevented by a highly effective antioxidant defense system including enzymatic (Cu/Zn superoxide dismutase, catalase, glutathione peroxidase, and peroxiredoxins) and non-enzymatic (glutathione) scavengers [reviewed in Jeney et al. (9)]. In contrast, outside RBCs, Hb is prone to oxidation, giving rise to the formation of different Hb oxidation products [metHb (Fe^3+^), ferrylHb (Fe^4+^ = O^2–^)] and subsequent release of heme. The formed high-valence (Fe^4+^) iron compounds are reactive intermediates and decay quickly via intramolecular electron transfer between the ferryl iron and specific amino acid residues of the globin chains resulting in the formation of globin radicals (10). Then the termination of the reaction occurs when these globin radicals react with each other leading to the formation of covalently cross-linked Hb multimers [reviewed in Jeney et al. (9)]. Covalently cross-linked oxidized Hb forms have been detected in different biological samples including plasma following intravascular hemolysis as well as in human complicated atherosclerotic lesions with intraplaque hemorrhage (11, 12).

Oxidized Hb forms (metHb, ferrylHb) and labile heme exert diverse pro-oxidant and pro-inflammatory activities toward different cell types including endothelial cells (ECs). As pro-oxidants, they induce lipid peroxidation and sensitize ECs to oxidant-mediated killing (13, 14). Heme induces toll-like receptor 4 (TLR4) activation and subsequent upregulation of adhesion molecules VCAM-1, ICAM-1, and E-selectin in ECs (15).

Besides heme, ferrylHb but not Hb or metHb induces upregulation of adhesion molecules in ECs, but interestingly, this response is not dependent on TLR4 activation (16). Increased endothelial permeability contributes to inflammatory cell extravasation upon hemolysis, and previous studies showed that ferrylHb and free heme induce the loss of endothelial integrity (16–20).

The goal of the present study was to perform a qualitative and quantitative analysis of the Hb content of human CSF samples obtained from premature infants following IVH with a special interest in detecting ferrylHb/covalently cross-linked Hb species. We also aimed to investigate the pro-oxidant and pro-inflammatory effects of these Hb forms on human brain microvascular endothelial cells (HBECs). We determined the levels of inflammatory markers in post-IVH CSF samples and correlated their values to the heme content of CSF samples to further understand the role of heme in triggering the inflammatory response following IVH. We believe that a better understanding of the molecular mechanism of the post-IVH inflammatory response is critical in tailoring therapeutic tools to avoid these infants from the development of the life-long neurological effects of IVH.

## Materials and Methods

### Materials

Reagents were purchased from Sigma-Aldrich (St. Louis, MO, United States) unless otherwise specified.

### Patient Selection and CSF Collection

In this study, we used the leftover of CSF samples that were collected by spinal tap or ventricular reservoir puncture for diagnostic purposes at the Department of Neurosurgery, University of Debrecen. Preterm infants (*n* = 20) diagnosed with grade III IVH with a mean gestational age at birth of 27.9 ± 2.2 weeks were involved in the study. CSF samples were collected at 26.6 ± 16.4 days after the onset of IVH. No CSF was obtained exclusively for inclusion in this study. Within 30 min of collection, CSF samples were centrifuged (2,000 *g*, 4°C, 15 min), and supernatants were stored aliquoted at −70°C until analysis. The procedures were approved by the Scientific and Research Ethics Committee of the University of Debrecen and the Ministry of Human Capacities under the registration number of 1770-5/2018/EÜIG. Parental consent forms were signed by the parents of the infants involved in this study.

### Determination of Hb, metHb, ferrylHb, Total Heme, Free Heme, and Bilirubin Levels in CSF

The absorbance spectra (250–700 nm) of CSF samples were taken with a spectrophotometer (NanoDrop 2000, Thermo Fisher Scientific, MA, United States). Concentrations of Hb, metHb, and ferrylHb were calculated from the absorbance values measured at 541, 576, and 630 nm, using the absorption coefficients and equations determined previously by Meng and Alayash (21). The total heme concentration of CSF samples was determined by using a QuantiChrom Heme Assay Kit (Gentaur Ltd., London, United Kingdom) according to the manufacturer’s instructions. Concentration of non-Hb bound heme was calculated by the following equation: [free heme] = [total heme] – [Hb-heme] – [metHb-heme] – [ferrylHb]. Bilirubin levels in CSF samples were measured by a colorimetric assay on a Cobas 6000 analyzer (Roche Diagnostics, Mannheim, Germany).

### Cell Culture

HBEC cell line was purchased from ATCC (CRL-3245, Manassas, VA, United States). Cells were cultured in Media 199, supplemented with 10% fetal bovine serum (Gibco, Waltham, MA, United States), 40 μg/ml EC growth supplement, and 1% penicillin/streptomycin in 5% CO_2_ humidified atmosphere at 37°C. HBECs were used at passages 5 and 8.

### Hemoglobin Preparation

We prepared Hb, metHb, and ferrylHb from fresh blood obtained from healthy volunteers as described in detail in our previous work (12). Briefly, Hb was isolated from fresh blood drawn from healthy volunteers using ion-exchange chromatography on a DEAE Sepharose CL-6B column. metHb was generated by incubation (30 min, 25°C) of purified Hb with a 1.5-fold molar excess of K_3_Fe(CN)_6_ over heme. FerrylHb was obtained by incubation (1 h, 37°C) of Hb with a 10:1 ratio of H_2_O_2_ to heme. The ferryl state of iron is highly unstable and therefore ferrylHb transiently forms. During stabilization of ferryl iron, different chemically heterogeneous oxidized Hb molecules are formed, which we refer to as ferrylHb to reflect rather the way of their formation than their actual oxidation status. After oxidation, both metHb and ferrylHb were dialyzed against saline (three times for 3 h at 4°C) and concentrated using Amicon Ultra centrifugal filter tubes (10,000 MWCO, Millipore Corp., Billerica, MA, United States). Aliquots were snap-frozen in liquid nitrogen and stored at −70°C until use.

### Cell Viability Assay

Confluent HBECs grown in 96-well tissue-culture plates were washed twice with Hank’s Balanced Salt Solution (HBSS) and exposed to heme and different Hb species (Hb, metHb, or ferrylHb at a concentration of 10–100 μmol/L heme group) for 24 h. Then cells were washed with HBSS, and 100 μl of 3-[4,5-dimethylthiazol-2-yl]-2,5-diphenyl-tetrazolium bromide (MTT) (0.5 mg/ml) solution in HBSS was added. After a 4-h incubation, the MTT solution was removed, formazan crystals were dissolved in 100 μl of dimethyl sulfoxide, and optical density was determined at 570 nm.

### Endothelial Cell Monolayer Integrity Assay

The electric cell-substrate impedance sensing (ECIS) method was used to measure the endothelial monolayer integrity. HBECs were cultured in 8-well electrode arrays (8W 10E, Applied BioPhysics Inc., Troy, NY, United States). After reaching confluence, cells were treated with different Hb species (Hb, metHb, and ferrylHb at a concentration of 50 μmol/L heme), and the complex impedance spectrum was monitored with an ECIS Zθ instrument (Applied BioPhysics Inc., Troy, NY, United States) for 4 h. Results are shown as the difference between monolayer resistance at 4,000 Hz at 0 time point and 4 h.

### Quantitative Real-Time PCR

Total RNA was isolated from HBECs using TRizol (RNA-STAT60, Tel-Test Inc., Friendswood, TX, United States) according to the manufacturer’s protocol. Two micrograms of RNA was reverse-transcribed to cDNA with a High-Capacity cDNA Reverse Transcription Kit (Applied Biosystems, Waltham, MA, United States). PCR was performed using iTaq Universal Probes Supermix (BioRad Laboratories, Hercules, CA, United States) and predesigned primers and probes (TaqMan^®^ Gene Expression Assays) VCAM-1 (Hs01003372), ICAM-1 (Hs00164932), HO-1 (Hs01110250), IL-8 (Hs00174103), and GAPDH (Hs0278624). Relative mRNA expressions were calculated with the ΔΔCt method using GAPDH as an internal control.

### Intracellular ROS Measurement

ROS production was monitored by using the 5-(and-6)-chloromethyl-2’,7’-dichlorodihydrofluorescein diacetate, acetyl ester (CM-H2DCFDA) assay (Life Technologies, Carlsbad, CA, United States). Confluent HBECs were exposed to the Hb forms Hb, metHb, ferrylHb, and heme (10, 25, 50, and 100 μmol/L heme) for 4 h in M199 media supplemented with 1% FBS. Then cells were loaded with CM-H2DCFDA (10 μmol/L, 30 min, at 37°C in the dark), followed by three washes with HBSS. Fluorescence intensity was measured every 30 min for 4 h applying 488-nm excitation and 533-nm emission wavelengths.

### Western Blot

Whole cell lysates (20 μg/lane) or CSF samples (5 μl/lane) were resolved on 10% SDS-PAGE, then blotted onto a nitrocellulose membrane (Amersham Proton 1060003, GE Healthcare, Chicago, IL, United States). Western blot was performed with the use of the following polyclonal antibodies: anti-HO-1 antibody (70081, Cell Signaling Technology Inc., Danvers, MA, United States) at a concentration of 50 ng/ml and anti-VCAM-1 antibody (Sc-8304, Santa Cruz Biotechnology, Inc., Dallas, TX, United States) at a concentration of 1 μg/ml. We used peroxidase labeled anti-rabbit IgG (NA931, Amersham Bioscience, Piscataway, NJ, United States) as a secondary antibody at a concentration of 20 ng/ml. For Hb detection, we used an HRP-conjugated goat antihuman Hb polyclonal antibody (ab19362-1, Abcam Plc., Cambridge, United Kingdom) at a concentration of 0.1 μg/ml. Antigen–antibody complexes were visualized with the horseradish peroxidase chemiluminescence system (Amersham Biosciences Corp., Piscataway, NJ, United States). Chemiluminescent signals were detected conventionally on an X-ray film or digitally by using a C-DiGit Blot Scanner (LI-COR Biosciences, Lincoln, NE, United States). After detection, the membranes were stripped and re-probed for β-actin using HRP-conjugated anti-β-actin antibody (Sc-47778, Santa Cruz Biotechnology, Inc., Dallas, TX, United States) at a concentration of 0.13 μg/ml. Blots were quantified by using the inbuilt software of the C-DiGit Blot Scanner (LI-COR Biosciences, Lincoln, NE, United States).

### Measurement of Soluble VCAM-1, ICAM-1, and IL-8 Levels in CSF Samples

To perform enzyme-linked immunosorbent assay (ELISA), CSF samples were first centrifuged at 10,000 *g* for 1 min. Soluble VCAM-1 and ICAM-1 protein concentrations were quantitatively measured by ELISA as described in the manufacturer protocol (R&D Systems, Minneapolis, MN, United States). Levels of IL-8 were determined by ELISA (BD OptEIA; BD Biosciences, San Diego, CA, United States).

### Statistical Analysis

Results are expressed as mean ± SD. At least three independent experiments were performed for all *in vitro* studies. Statistical analyses were performed with GraphPad Prism software (version 8.01, San Diego, CA, United States). Comparisons between more than two groups were carried out by ordinary one-way ANOVA followed by *post hoc* Tukey’s multiple-comparisons test. We applied one-way ANOVA followed by Dunnett’s *post hoc* test when experimental groups were compared to a control. A value of *p* < 0.05 was considered significant. To measure the strength of the association between two variables, we performed Pearson’s correlation analysis. A strong positive correlation was defined as a value of Pearson’s correlation coefficient (*r*) > 0.4.

## Results

### Time-Dependent Accumulation of Different Oxidized Hb Forms, Free Heme, and Bilirubin in Post-IVH CSF Samples

In this study, we have analyzed 20 CSF samples that were collected by spinal tap or ventricular reservoir puncture from preterm infants diagnosed with grade III IVH. The main characteristics of the patients are summarized in Table 1. All patients were preterm infants with a median gestational age of 28 weeks at delivery (Table 1). The mean birth weight of the infants was 1,094 ± 282 g (Table 1). Out of the 20 infants, 10 did not receive steroid prophylaxis, and 8 obtained partial steroid prophylaxis. In addition, 14 infants were born via Cesarean section, 18 developed hydrocephalus, and 2 of them died before 6 months of age (Table 1).

**TABLE 1:** Characteristics of the patients.

Characteristic	Grade III IVH (*n* = 20)
**Male sex** - no./total no.	11/20
**Gestational age at delivery** Median - week Distribution 23 week 0 days to 25 week 6 days 26 week 0 days to 27 week 6 days 28 week 0 days to 29 week 6 days 30 week 0 days to 31 week 6 days	28 4 4 6 6
**Birth weight** Mean ± s.d. - g Distribution ≥500 to <750 g ≥750 to <1000 g ≥1000 to <1250 g ≥1250 to <1500 g	1094 ± 282 3 6 4 7
**Apgar 5 min** Median Distribution 0–3 4–6 7–8 9–10	5 4 13 3 0
**Apgar 10 min** Median Distribution 0–3 4–6 7–8 9–10	8 0 7 9 4
**Steroid prophylaxis**- yes/partial/no	2/8/10
**Multiply pregnancy**- no./total no.	5/20
**Cesarean section delivery**- no./total no.	14/20
**Hydrocephalus**- no./total no.	18/20
**Death**- no./total no.	2/20
**Timing of the CSF samples [days after the onset of IVH, (*n*)]**	14(1), 15(1), 16(1), 17(2), 19(1), 20(2), 21(4), 24(1), 25(1), 28(1), 32(1), 45(1), 60(3)

*CSF samples were collected from premature infants (*n* = 20) diagnosed with grade III IVH.*

**FIGURE 1:**
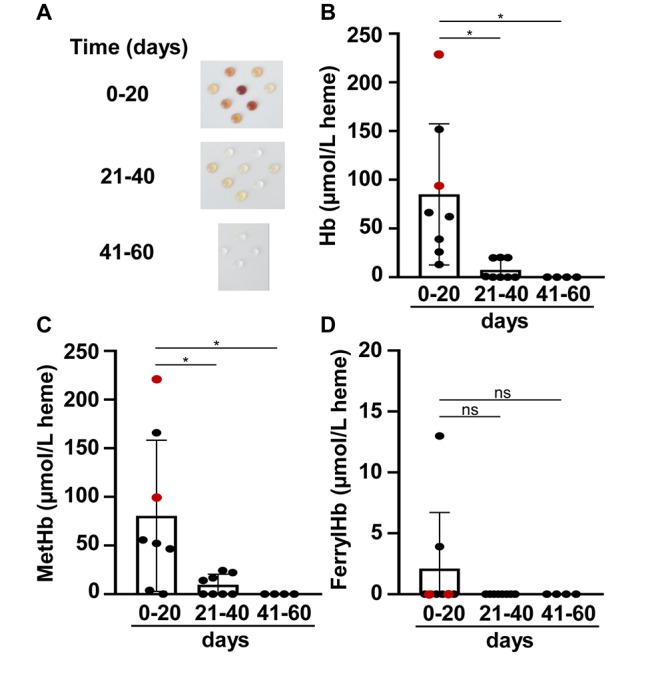
Time-dependent accumulation of Hb, metHb, and ferrylHb in post-IVH CSF samples. CSF samples (*n* = 20) were obtained from preterm infants diagnosed with grade III IVH at different time intervals after the onset of IVH. **(A)** Physical appearance of CSF samples obtained at different time intervals after the onset of IVH (days 0–20, 21–40, 41–60). **(B–D)** Concentrations of Hb, metHb, and ferrylHb were quantified spectrophotometrically in CSF samples. Closed circles represent individual samples, red circles represent patients that died before 6 months of age, and bars represent mean ± SD values. *P*-values were calculated using one-way ANOVA followed by Tukey’s multiple comparison analysis. *p < 0.05.

Because CSF samples were taken for diagnostic purposes, we obtained CSF samples at different time points after the onset of IVH (days 14–60, mean: 27.6 ± 15.6 days, median: 21 days). Based on the elapsed time between the onset of IVH and CSF sampling, we divided the samples into three groups, 0–20 days, 21–40 days, and 41–60 days. CSF samples obtained at different time intervals after the onset of the IVH had different colors, i.e., 0–20 days CSF samples had brownish discoloration, 21–40 days CSF samples were yellowish, whereas 41–60 days CSF samples were colorless similar to a normal CSF specimen (Figure 1A). To evaluate Hb, metHb, and ferrylHb concentrations in CSF, we took the visible absorption spectra of the samples and calculated Hb concentrations with the use of molar extinction coefficients as determined previously (21). The Hb levels of CSF samples obtained 0–20 days after the onset of IVH showed a big variation from 13.08 up to 228.12 μmol/L, with an average of 85.04 ± 72.38 μmol/L. The Hb concentration in CSF samples obtained at later time points, i.e., 21–40 days after IVH onset, was significantly lower (7.61 ± 10.32 μmol/L), and Hb was undetectable in CSF samples collected 41–60 days following IVH (Figure 1B).

One-electron oxidation of Hb leads to the formation of metHb. Similarly to that of Hb, we found high amounts of metHb in CSF samples obtained at 0–20 days (80.51 ± 77.65 μmol/L), which decreased gradually during the study period (Figure 1C). On average, metHb concentration was 9.65 ± 10.77 μmol/L in CSF samples obtained at 21–40 days after the onset of IVH and was below the detection limit in CSF samples collected after 41–60 days of IVH (Figure 1C). Two-electron oxidation of Hb by peroxides, for instance, could lead to the formation of ferrylHb. Interestingly, we could hardly detect any ferrylHb in the CSF samples, which could be explained by the highly unstable nature of this Hb species (Figure 1D).

**FIGURE 2:**
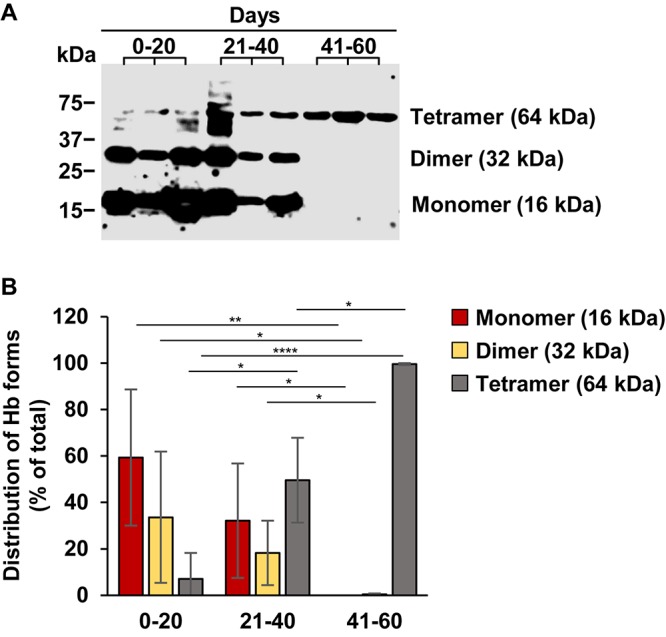
Time-dependent accumulation of covalently cross-linked globin multimers in post-IVH CSF samples. Hb contents of CSF samples (*n* = 20) obtained from preterm infants diagnosed with grade III IVH at different time intervals after the onset of IVH were analyzed by western blot. **(A)** Representative western blot is shown. **(B)** Densitometric analysis of western blots of all samples (*n* = 20) was performed and percentage of globin monomers, dimers, and tetramers (Hb) is presented as mean ± SD. *P*-values were calculated using one-way ANOVA followed by Tukey’s multiple comparison analysis. *p < 0.05, **p < 0.01, ****p < 0.001.

FerrylHb is a reactive intermediate that decays quickly via intramolecular electron transfer between the ferryl iron and specific amino acid residues of the globin chains. In this reaction, globin radicals are produced, which are still unstable and react with each other to get stabilized via the formation of covalent bonds between the globin subunits (9). Next we addressed whether this occurs following IVH in the CSF. We have analyzed all the CSF samples by western blot under reducing conditions and detected Hb forms with different molecular weights that corresponded as globin monomers (16 kDa), dimers (32 kDa), and tetramers (Hb) (64 kDa). A representative western blot is shown in Figure 2A. Densitometric analysis of the western blots revealed that in CSF samples obtained at 0–20 days after the onset of IVH contained predominantly globin monomers (59.3 ± 29.3% of total Hb), less globin dimers (33.6 ± 28.2% of total Hb), and very low amounts of Hb tetramers (7.03 ± 11.1% of total Hb) (Figures 2A,B). Interestingly, we observed a shift toward the formation of globin dimers and tetramers in CSF samples obtained at 21–40 days post-IVH (Figures 2A,B). This change becomes highly remarkable in CSF samples obtained after 41–60 days of IVH, as these samples contained neither monomers nor dimers but contained Hb tetramers (Figures 2A,B).

**FIGURE 3:**
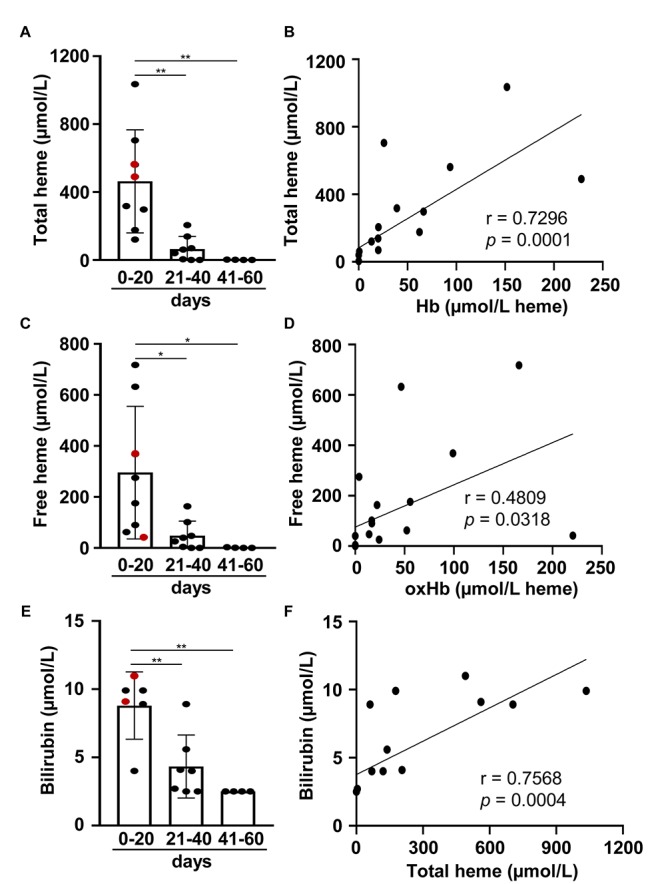
Time-dependent accumulation of total heme, free heme, and bilirubin in post-IVH CSF samples. CSF samples (*n* = 20) were obtained from preterm infants diagnosed with grade III IVH at different time intervals (days 0–20, 21–40, 41–60) after the onset of IVH. **(A,C,E)** Total heme, free heme, and bilirubin levels of CSF samples were determined. Closed circles represent individual samples, red circles represent patients that died before 6 months of age, and bars represent mean ± SD. *P*-values were calculated using one-way ANOVA followed by Tukey’s multiple comparison analysis. *p < 0.05, **p < 0.01. **(B,D,E)** Correlation between **(B)** Hb and total heme concentrations, **(D)** oxidized Hb forms (MHb + FHb) and free heme concentrations, and **(F)** total heme and bilirubin concentrations in post-IVH CSF samples (*n* = 20) is shown. R represents Pearson’s correlation coefficient.

Hb oxidation can lead to the dissociation of the heme group from the globin giving a rise in the formation of non Hb-bound (free) heme. To see whether this occurred following IVH, first we determined total heme levels in CSF samples. Total heme levels were very high in CSF obtained at 0–20 days after the onset of IVH ranging from 120.02 up to 1,035.27 μmol/L with an average of 463.01 ± 303.39 μmol/L (Figure 3A). Total heme levels were significantly lower in CSF samples obtained at 21–40 days after the onset of IVH (64.98 ± 73.50 μmol/L) and was below 1 μmol/L in CSF samples collected 41–60 days post-IVH (Figure 3A). Correlation analysis between Hb and total heme levels revealed a very strong linear correlation (*r* = 0.7296) between the two variables, suggesting that Hb is the major source of heme in CSF as we expected (Figure 3B). Next we calculated the concentration of free heme as described in the methods. Free heme concentrations were high in CSF collected at 0–20 days after the onset of IVH ranging from 41.99 to 717.39 μmol/L with an average of 295.34 ± 259.80 μmol/L (Figure 3C). Free heme levels were significantly lower in CSF samples obtained at 21–40 days after the onset of IVH (47.73 ± 57.50 μmol/L) and was below 1 μmol/L in CSF samples collected 41–60 days post-IVH (Figure 3C). We looked at the correlation between the concentration of oxidized Hb (metHb + ferrylHb) and free heme and found a strong positive correlation (*r* = 0.4809) between the two variables, which supports the concept that oxidized Hb forms can release their heme prosthetic groups (Figure 3D). Additionally, we measured the concentration of bilirubin, one of the end-products of heme catabolism in CSF samples. We found that bilirubin levels were the highest (8.8 ± 2.47 μmol/L) in CSF collected at 0–20 days after the onset of IVH, and then it gradually decreased in time (Figure 3E). We found that bilirubin levels strongly correlated to the total heme levels in post-IVH CSF samples (*r* = 0.7568) (Figure 3F).

### Pro-oxidant and Pro-inflammatory Effects of Hb Forms and Free Heme Toward Human Brain Microvascular Endothelial Cells

IVH in preterm infants leads to systemic inflammation characterized by increased levels of pro-inflammatory cytokines and cellular adhesion molecules. ECs play a critical role in the pro-inflammatory responses, and Hb oxidation products have been shown to be implicated in diverse hemolysis-associated sterile inflammatory reactions. Therefore, next we have addressed the pro-oxidant and pro-inflammatory effects of the different Hb oxidation products that were identified in post-IVH CSF samples toward HBECs.

**FIGURE 4:**
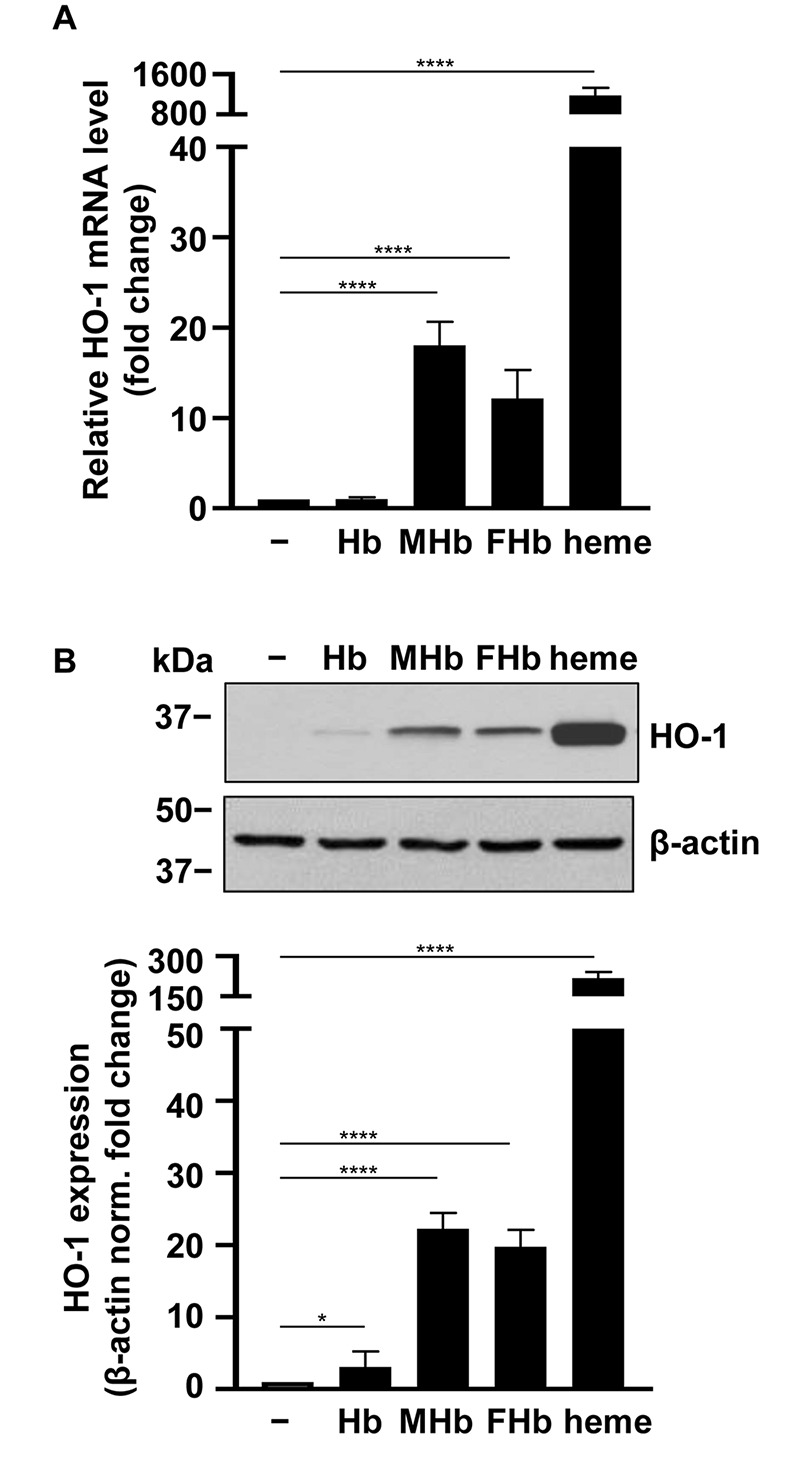
Induction of HO-1 mRNA and protein expression by different Hb forms in HBECs. Confluent HBECs (P5-8) were exposed to vehicle, Hb, MetHb (MHb), ferrylHb (FHb), and heme (25 μmol/L heme group) in 1% FBS. **(A)** Relative mRNA expression (4 h, mean ± SD) of HO-1 normalized to GAPDH from three independent experiments performed in triplicates. **(B)** HO-1 protein levels (16 h) were analyzed by western blot from whole cell lysates. Membranes were re-probed for β-actin. Representative blots of three independent experiments are shown. Densitometry analysis (mean ± SD) of three independent experiments. **(A,B)** *P*-values were calculated using one-way ANOVA followed by Dunnett’s *post hoc* analysis. *p < 0.05 and ****p < 0.001.

First we have addressed whether the different Hb forms induce heme oxygenase-1 (HO-1), the stress-responsive inducible enzyme that catalyzes heme degradation upon heme overload conditions. We exposed HBECs to Hb, metHb (MHb), ferrylHb (FHb), and free heme (25 μmol/L heme group). Oxidized Hb forms, i.e., metHb and ferrylHb, induced an 18.2 ± 2.6-fold and a 12.1 ± 3.1-fold elevation of HO-1 mRNA levels (4 h), respectively (Figure 4A). In contrast, native Hb did not cause an elevation of HO-1 mRNA (Figure 4A). Free heme was a highly more potent inducer of HO-1 in HBECs in comparison with metHb and ferrylHb triggering a more than 1,000-fold elevation of the HO-1 mRNA level after a 4-h exposure (Figure 4A). In parallel with the changes of the HO-1 mRNA level, both metHb and ferrylHb induced an about 20-fold elevation of the HO-1 protein expression, whereas free heme increased the HO-1 expression by about 200-fold (Figure 4B).

**FIGURE 5:**
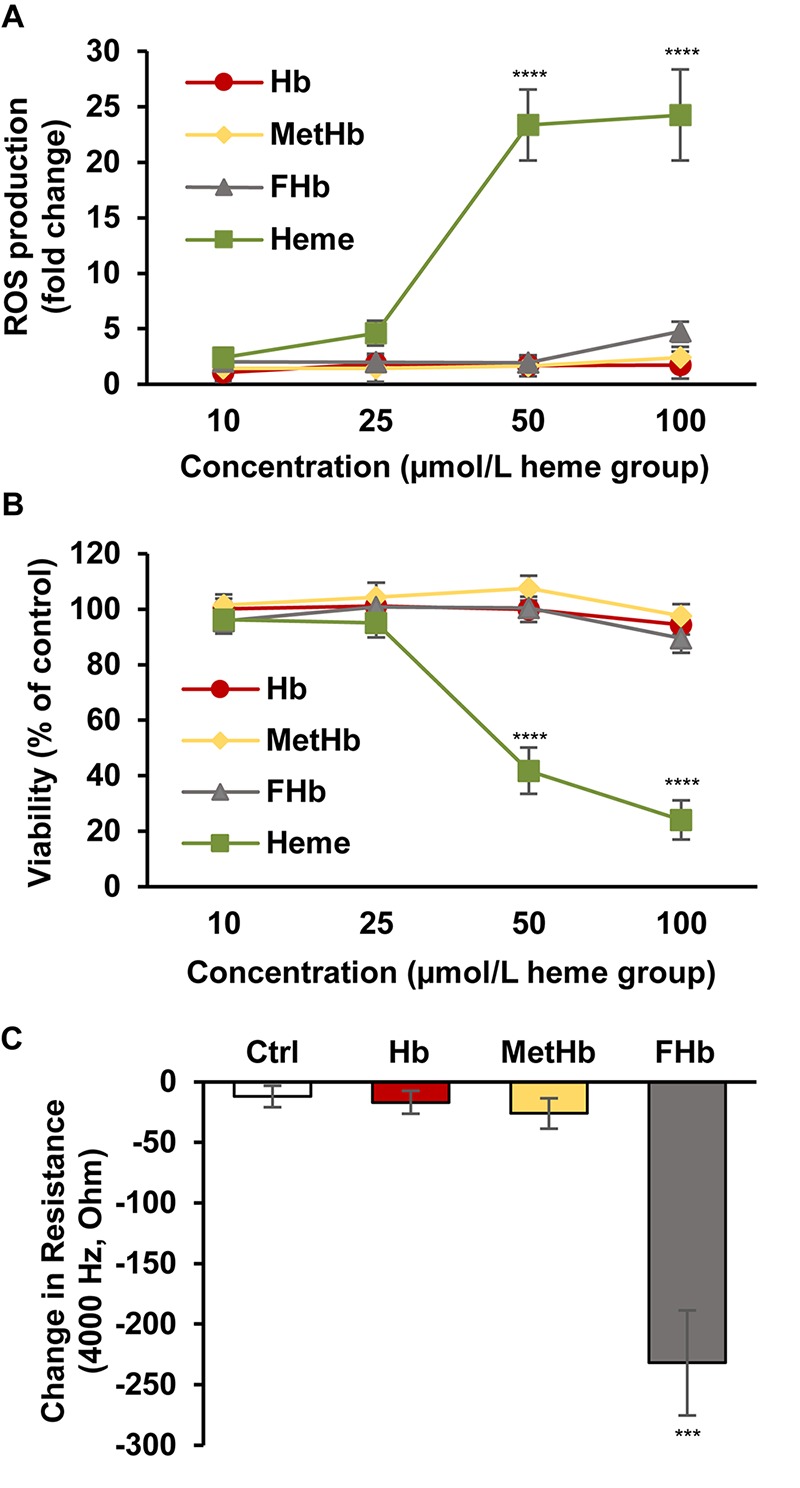
Induction of ROS production and cell death by different Hb forms in HBECs. **(A)** Confluent HBECs (P5-8) were exposed to vehicle, Hb, MetHb, ferrylHb (FHb), and heme (10, 25, 50, 100 μmol/L heme group) in 1% FBS for 4 h, then ROS production was monitored by DCFDA assay for an additional 4 h. Graph shows ROS production (mean ± SD) at 4-h time point from three independent experiments performed in quadruplicates. **(B)** Confluent HBECs (P5-8) were exposed to vehicle, Hb, MetHb, ferrylHb (FHb), and heme (10, 25, 50, 100 μmol/L heme group) in 1% FBS and cellular viability (24 h) was assessed by MTT assay. Graph shows cell viability as a percentage of viability of vehicle-treated cells (mean ± SD) from three independent experiments performed in quadruplicates. **(C)** HBECs cultured in 8-well ECIS plates were exposed to Hb, metHb, and ferrylHb (FHb) (50 μmol/L heme group) in 1% FBS containing media. The complex impedance spectrum was monitored with an ECIS Zθ instrument for 4 h. Change in resistance (mean ± SD) was calculated based on the difference between monolayer resistance at 4,000 Hz at 0 time point and 4 h from three independent experiments performed in triplicates. **(A–C)** *P*-values were calculated using one-way ANOVA followed by Tukey’s multiple comparison analysis. ***p < 0.005, ****p < 0.001.

Heme is a catalyst of the Fenton reaction and therefore is implicated in the sustained production of reactive oxygen species (ROS) under hemolytic conditions. Next we investigated whether the Hb oxidation products found in post-IVH CSF samples induce ROS production in HBECs. HBECs were treated with Hb, metHb, ferrylHb, and heme (10, 25, 50, 100 μmol/L heme group) for 4 h, and ROS formation was measured as described in the methods. Heme at concentrations of 50 and 100 μmol/L induced substantial production of ROS (Figure 5A). In contrast, neither native Hb nor oxidized Hb forms (metHb and ferrylHb) increased ROS production in HBECs (Figure 5A). To see whether Hb oxidation products induce HBEC death, we exposed the cells to Hb, metHb, ferrylHb, and free heme (10, 25, 50, 100 μmol/L heme group) for 24 h. Heme at concentrations of 50 and 100 μmol/L induced a substantial reduction in cell viability (Figure 5B). On the contrary, cell viability was unaffected by the Hb forms, even when they were applied at the highest concentration (Figure 5B). A previous study on human umbilical vein ECs showed that oxidized Hb increased endothelial monolayer permeability (16). Therefore, we investigated whether the Hb forms impair HBEC monolayer integrity. We exposed HBECs to Hb, metHb, and ferrylHb (50 μmol/L heme group) and measured the changes of monolayer resistance over a 4-h period of time (Figure 5C). HBEC monolayer integrity was unaffected by native Hb or metHb treatment. In contrast, ferrylHb treatment largely impaired HBEC monolayer integrity (Figure 5C).

**FIGURE 6:**
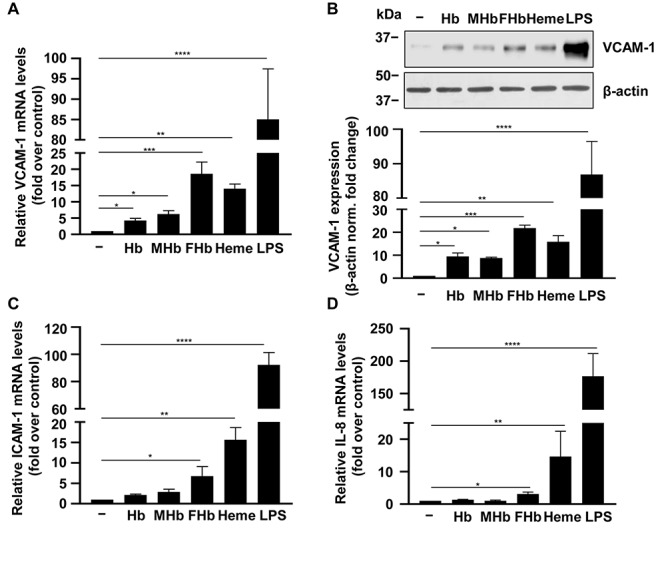
Pro-inflammatory effects of different Hb forms in HBECs. **(A–D)** Confluent HBECs (P5-8) were exposed to vehicle, Hb, metHb (MHb), ferrylHb (FHb), heme (25 μmol/L heme group), and LPS (100 ng/ml) in 1% FBS. Relative mRNA expression (4 h, mean ± SD) of VCAM-1, ICAM-1, and IL-8 normalized to GAPDH from three independent experiments performed in triplicates are shown. **(B)** VCAM-1 protein levels (16 h) were analyzed by western blot from whole cell lysate. Membranes were re-probed for β-actin. Representative blots of three independent experiments are shown. Densitometry analysis (mean ± SD) of three independent experiments. **(A–D)** *P*-values were calculated using one-way ANOVA followed by Dunnett’s *post hoc* analysis. *p < 0.05, **p < 0.01, ***p < 0.005, ****p < 0.001.

Heme and oxidized Hb forms have been shown to be implicated in the immune response in hemolytic diseases. More specifically, heme and ferrylHb have been shown to upregulate the expression of cellular adhesion molecules including VCAM-1 and ICAM-1 in human umbilical vein ECs. Here we treated HBECs with Hb, metHb, ferrylHb, and heme (25 μmol/L heme group) and measured mRNA expressions (4 h) of VCAM-1, ICAM-1, and the pro-inflammatory cytokine IL-8 (Figures 6A–D). We used LPS (100 ng/ml) as a positive control in these experiments. Free heme and ferrylHb triggered marked elevations of VCAM-1 mRNA (∼15–20-fold), whereas the effect of native Hb and metHb was milder inducing about 5-fold increases of VCAM-1 (Figure 6A). We observed a similar trend on the protein level, namely, ferrylHb and free heme were more potent in inducing VCAM-1 expression than native Hb and metHb (Figure 6B). Additionally, free heme and ferrylHb but not native Hb and metHb induced elevations of ICAM-1 and IL-8 mRNA levels (Figures 6C,D). We have to note that the bioavailable heme concentrations in these experiments were much lower than 25 μmol/L due to the presence of specific (Hx) and non-specific (albumin) heme-binding proteins present in the serum, which was applied at 1% in these experiments.

### Correlations Between Levels of Heme and Pro-inflammatory Markers in Post-IVH CSF Samples

**FIGURE 7:**
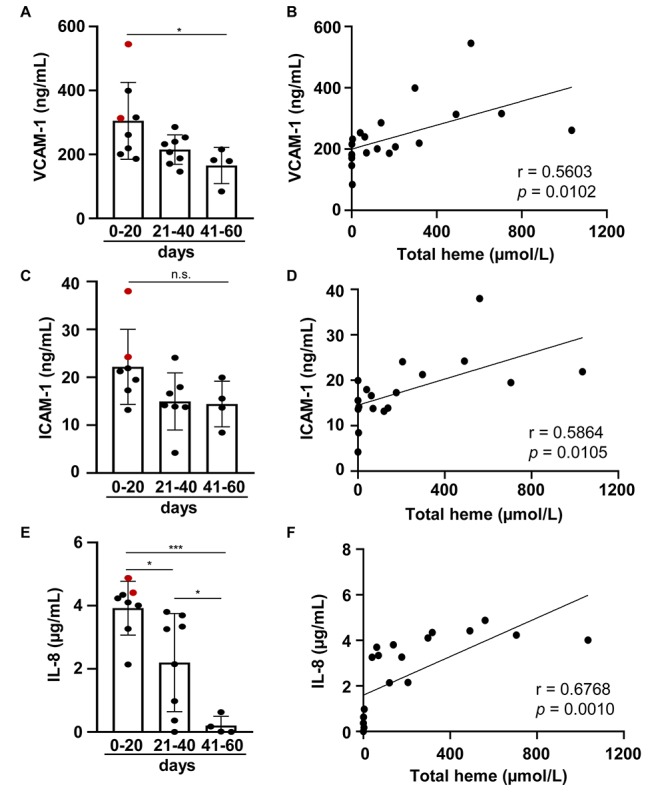
Level of soluble VCAM-1, soluble ICAM-1, and IL-8 in post-IVH CSF samples. CSF samples (*n* = 20) were obtained from preterm infants diagnosed with grade III IVH at different time intervals after the onset of IVH. **(A,C,E)** Soluble VCAM-1, ICAM-1, and IL-8 levels of CSF samples obtained at different time intervals after the onset of IVH (days 0–20, 21–40, 41–60) were determined by ELISA in duplicates. Closed circles represent individual samples, red circles represent patients that died before 6 months of age, and bars represent mean ± SD. *P*-values were calculated using one-way ANOVA followed by Tukey’s multiple comparison analysis. Correlation between **(B)** total heme and soluble VCAM-1, **(D)** total heme and soluble ICAM-1, and **(F)** total heme and IL-8 levels in post-IVH CSF samples is shown. R represents Pearson’s correlation coefficient. *p < 0.05, ***p < 0.005.

Previous studies showed that the levels of pro-inflammatory markers including soluble adhesion molecules and inflammatory cytokines are elevated in post-IVH CSF samples. Our *in vitro* data suggested that Hb-derived heme may play a critical role in the induction of the pro-inflammatory response. To address this question, we measured VCAM-1, ICAM-1, and IL-8 levels in post-IVH CSF samples (*N* = 20). VCAM-1 levels were the highest in CSF samples obtained between 0 and 20 days after the onset of IVH (305.11 ± 120.12 ng/ml) (Figure 7A). Comparing to these samples, VCAM-1 levels were significantly lower in CSF samples obtained at 41–60 days after the onset of IVH (165.31 ± 56.51 ng/ml) (Figure 7A). Then we analyzed whether there is a correlation between heme and VCAM-1 levels in the post-IVH CSF samples, and we found a strong linear correlation between the two variables (*r* = 0.5603) (Figure 7B). Next, we determined the level of soluble ICAM-1 in post-IVH CSF samples. We observed a decreasing trend of ICAM-1 levels, but the differences were not significant (Figure 7C). On the other hand, ICAM-1 levels correlated strongly (*r* = 0.5864) with total heme concentrations in post-IVH CSF samples (Figure 7D). Finally, we have measured the level of the pro-inflammatory cytokine IL-8 in the post-IVH CSF samples. We found that IL-8 levels were the highest in CSF samples obtained at 0–20 days after the onset of IVH (3.92 ± 0.85 μg/ml), and then we observed a gradual decrease in IL-8 levels at 21–40 and 41–60 days after the onset of IVH resulting in 2.19 ± 1.5 and 0.2 ± 0.29 μg/ml IL-8 concentrations, respectively (Figure 7E). Additionally, we found a strong positive correlation between total heme concentration and IL-8 levels in post-IVH CFS samples (*r* = 0.6768) (Figure 7F).

## Discussion

IVH is a frequent complication of prematurity that associates with high neonatal mortality and increased risk of neurodevelopmental impairment in the surviving infants (1–4). It is known for a long time that inflammation plays a critical role in the pathophysiology of IVH-induced brain damage; however, the molecular mechanism by which IVH stimulates the inflammatory response is not fully understood. Extravasation of blood into the intraventricular space triggers a cascade of events including the release of various vasoactive and pro-inflammatory molecules from blood and the vascular system [reviewed in Sercombe et al. (22)].

In this study, we performed a qualitative and quantitative analysis of Hb content of human CSF samples obtained from premature infants following IVH at different time points after the onset of IVH to understand the kinetics of Hb release, oxidation, and clearance. We investigated the pro-oxidant and pro-inflammatory effects of the identified Hb forms on HBECs to extend our understanding of the particular roles that these species may play in the neuroinflammatory response following IVH. We measured the levels of pro-inflammatory markers in post-IVH CSF samples at different time points after the onset of IVH to explore the kinetics of the inflammatory response and investigated whether the inflammatory response correlates to the extent of hemolysis.

We showed that after IVH Hb is released into the CSF and Hb oxidation occurs leading to the formation of metHb, ferrylHb, and covalently cross-linked oxidized globin multimers. Previous studies showed that cell-free Hb and Hb metabolites are present in CSF following different types of intracranial hemorrhage including IVH (23–25). Particularly, metHb that is produced in a one-electron oxidation of Hb was detected in CSF samples obtained following IVH in preterm infants as well as in an experimental rabbit model of IVH (23). On average, the metHb level in the CSF samples obtained between days 0 and 20 after the onset of IVH was 80.51 ± 77.65 μmol/L, which is in good agreement with the previously reported level of metHb in CSF (∼ 40 μmol/L) on day 3 post-IVH in a rabbit model (23).

Peroxides trigger a two-electron oxidation of Hb, leading to the generation of ferrylHb in which the oxidation state of iron is +4. This unstable form of oxidized Hb was detected in human blood under physiologic and pathophysiologic conditions, but whether this form is produced following IVH has never been addressed (26–28). We could detect ferrylHb only in two out of eight CSF samples obtained between days 0 and 20 after the onset of IVH, which might be explained by the highly reactive nature of the ferryl iron.

High-valent iron in ferrylHb promotes the oxidation of definite amino acids of the globin chains and the subsequent intermolecular cross-linking of the globin subunits (9, 29, 30). Under *in vivo* conditions, these covalently cross-linked ferrylHb species were detected in different biological samples including plasma and urine following intravascular hemolysis as well as in human complicated atherosclerotic lesions with intraplaque hemorrhage (11, 12, 31). Here we showed for the first time that covalently cross-linked oxidized globin multimers, i.e., dimers and tetramers, are present in the CSF samples following IVH.

In the circulation, extracellular Hb is eliminated through the haptoglobin (Hp)–CD163 scavenger pathway (32). First extracellular Hb binds to Hp with extremely high affinity (Kd ∼ 10-12 mol/L) (33), then Hb–Hp complexes are internalized via CD163 receptors expressed on macrophages and monocytes (34). Regarding the CNS, Hp is present in CSF, but because of its low concentration, the Hb-binding capacity of CSF (∼100 μg Hb in adults) is far below the Hb-binding capacity of plasma (∼5 g Hb) (35). It has been shown that following IVH, Hb penetrates from the intraventricular space to the periventricular white matter and contributes to the development of IVH-associated brain injury.

Hb concentration of CSF samples obtained between days 0 and 20 after the onset of IVH showed a huge variation ranging from 13 up to 228 μmol/L with an average of 85 μmol heme groups/L. This corresponds to 10–530 mg of Hb in the 50-ml volume of CSF in the infants, and although we are lacking information about Hp levels in the CSF of premature infants, we assume that in most CSF samples, the level of cell-free Hb exceeds the Hb-binding capacity of CSF.

Once bound to Hp, Hb is protected from oxidation; therefore, the presence of oxidized Hb forms, i.e., metHb and ferrylHb in the post-IVH CSF samples support the idea that the level of Hb overwhelms the Hb binding capacity of Hp in CSF following IVH. Moreover, covalently cross-linked oxidized Hb multimers that formed upon Hb oxidation have limited affinity toward Hp; therefore, these species might bypass the homeostatic control of cell-free Hb (36).

In contrast to native Hb, MetHb as well as ferrylHb can release their heme prosthetic groups (13, 14, 37). Heme is a hydrophobic molecule, which allows its penetration through cell membranes by passive diffusion, although recently, cell surface and organelle-associated transporters were discovered to facilitate the movement of heme between the different cellular compartments [reviewed in Gozzelino (38)]. Heme has long been considered as a pro-oxidant molecule (39), and recently, its pro-inflammatory nature has been recognized as well (40). The pro-oxidant reactivity of heme relies on the ability of its iron atom to exchange electrons with a variety of substrates. For example, interaction of heme with H_2_O_2_ results in the formation of the highly reactive hydroxyl radical in the Fenton reaction. Consequently, heme sensitizes various cells to oxidant- or cytokine-mediated killing (41, 42). Regarding cells of the central nervous system (CNS), heme (5–40 μmol/L) has been shown to be cytotoxic toward astrocytes (43) and neurons (44), and sensitize oligodendrocytes to TNF-α mediated programmed cell death (42).

To see whether there is any non Hb-bound, so-called “free” heme in the post-IVH CSF samples, first we determined the total heme concentration in CSF (total heme = heme in all Hb forms + free heme), and then calculated the amount of free heme. We found a big individual variation in free heme levels in CSF samples obtained between days 0 and 20 after the onset of IVH ranging from 42 up to 717 μmol/L.

Intravascular free heme is eliminated by the CD91–heme–hemopexin scavenging system [reviewed in Smith and McCulloh (45)]. Hemopexin binds heme with the highest affinity of any known protein (46), inhibits its catalytic activity, and facilitates its removal via the CD91 receptor (47). The same CD91–heme–hemopexin route exists in the CNS and plays an important role in heme detoxification after subarachnoid hemorrhage in adults influencing the clinical outcome (48). We have no information whether the CD91–heme–hemopexin scavenging system has evolved fully in preterm infants or the heme removal capacity of such a system upon IVH, but in any case, we assume that the system is oversaturated and this contributes to the accumulation of free heme in the CSF after IVH.

Hb, metHb, total heme, and free heme levels were lower in CSF samples collected at later time points between days 21 and 40 after the onset of IVH in comparison to CSF samples collected during the first 20 days following IVH. This suggests that although the Hb and heme scavenging capacity of the CSF was overwhelmed right after the onset of IVH, a slow clearing procedure took place afterward. In CSF samples collected between days 41 and 60 after the onset of IVH, we could hardly detect heme in any form. CSF is renewed four to five times a day in adults, and this rate is considered to be even higher in neonates. CSF is cleared via the blood–CSF barrier through specific proteins that are expressed in the choroid plexus epithelial cells that provide transport of nutrients and ions into the CNS and removal of waste products and ions from the CSF. Clearance for Hb and its derivatives from CSF via the blood–CSF barrier could be an option following IVH, but this mechanism has not yet been characterized.

FerrylHb is stabilized by intermolecular electron transfer between the ferryl iron and an adjacent amino acid of the globin chain resulting in globin radicals. The formed globin-based radicals react with each other and form covalently cross-linked Hb multimers that we could detect in CSF samples. Interestingly, we found a clear shift toward the formation of higher multimers at later time points after the onset of IVH. In CSF obtained between days 21 and 40 after the onset of IVH, we found significantly more covalently cross-linked oxidized globin tetramers than in CSF collected earlier (days 0–20). Moreover, in the CSF samples collected between days 41 and 60 after the onset of IVH, we could detect exclusively the covalently cross-linked oxidized globin tetramers. Considering that we could not detect any heme in these samples, we concluded that these globin tetramers have already released their heme prosthetic groups. The presence of this form of Hb at days 41–60 after the onset of IVH suggests that there is no efficient mechanism in the CNS for the elimination of this oxidized Hb form.

The blood–brain barrier (BBB) prevents blood cells and pathogens from entering the brain parenchyma and regulates the transport of molecules between the plasma and the CNS (49). Intracerebral hemorrhage (ICH) is associated with BBB dysfunction, and several studies showed that blood components (e.g., thrombin, Hb, iron) play a major role in ICH-induced BBB dysfunction (50). The BMEC monolayer is an important component of the BBB, and in a critical manner contributes to the neuroinflammatory response mainly by inducing the leukocyte adhesion cascade to facilitate the transmigration of inflammatory cells into the CNS. Therefore, we investigated the effect of the identified Hb forms in post-IVH CSF samples on BMECs.

Free heme content of CSF obtained between days 0 and 20 after the onset of IVH was particularly high; only one out of eight CSF samples had a free heme content below 50 μmol/L, three samples had high free heme content (50–250 μmol/L), and four out of the eight CSF samples had very high free heme content that exceeded 250 μmol/L. We showed here that heme at the concentration of 50 μmol/L or higher causes BMEC death due to elevated production of ROS. On the other hand, neither native Hb nor the oxidized Hb forms triggered ROS production or EC death.

With the use of a guinea pig exchange transfusion model, it was previously shown that polymerized cell-free Hb triggers BBB disruption (51). Additionally, we showed earlier that ferrylHb induces intermolecular gap formation in HUVECs leading to decreased endothelial monolayer integrity (16). In agreement with the previous study, here we found that ferrylHb but not native Hb or metHb impairs BMEC monolayer integrity.

HO-1, the oxidative stress-responsive enzyme that catalyzes heme degradation, is induced following ICH in different cells of the CNS including astrocytes, microglia, and ECs (52). Here we showed that besides sublethal concentration of heme, oxidized Hb forms, i.e., metHb and ferrylHb induce HO-1 expression. HO-1 has antioxidant and anti-inflammatory actions and affords protection against programmed cell death and inhibits the pathogenesis of a variety of immune-mediated inflammatory diseases (42). In line with this notion, it was shown that the upregulation of HO-1 prevents the development of experimental cerebral malaria in mice and attenuates BBB disruption and neuroinflammation (53). These beneficial effects are considered to be mediated by the binding of carbon monoxide (CO) – the end-product of HO-1 activity – to Hb, preventing its oxidation and the generation of free heme (53).

Recent studies showed the beneficial effect of CO gas as well as CO releasing molecules (CORMs) in ICH. CO/CORM treatment alters the inflammatory response, attenuates vasospasm, improves neurobehavioral function, preserves the circadian rhythm, and overall reduces the severity of brain damage in experimental models of ICH (54–56). Further studies are needed to understand whether the beneficial effect of CO on ICH-induced brain damage relies on its ability to prevent oxidation of cell-free Hb and subsequent heme release.

Adhesion molecules mediate the inflammatory cell response to injury through adherence to the vascular endothelium, diapedesis through the endothelial barrier, and migration into the tissues. Normally, vascular endothelium is in a quiescent state characterized by low expression of adhesion molecules. In contrast, upon insult, endothelial dysfunction develops, characterized by elevated expression of adhesion molecules and pro-inflammatory cytokines. Brain hemorrhage triggers a local inflammatory response, and consequently, the levels of soluble adhesion molecules (i.e., E-selectin, ICAM-1, VCAM-1, and L-selectin) and inflammatory cytokines are elevated in the CSF of patients after subarachnoid hemorrhage (8, 57). IVH is followed by a systemic inflammatory response as well, and the extent of this response seems to be associated with white matter injury (7). ICAM-1 is a potential therapeutic target to attenuate cerebral vasospasm after subarachnoid hemorrhage (58).

Heme and oxidized Hb forms (i.e., metHb and ferrylHb) have been previously shown to induce adhesion molecules as well as pro-inflammatory cytokines (e.g., IL-6, IL-8, IL-1β) in human umbilical vein ECs (16, 59–61). Here we showed that heme and ferrylHb are the most potent inducers of the expression of adhesion molecules and IL-8 in BMECs, and that the levels of VCAM-1, ICAM-1, and IL-8 in CSF gradually decreased following the onset of IVH and correlated to total heme concentration of CSF.

Overall our study suggests that RBC destruction, Hb oxidation, and heme release are important pathogenic factors in IVH. On the other hand, this work also has some limitations. The study examined a very fragile group of patients: preterm babies with IVH grade III. The work is based on the examination of CSF samples obtained for diagnostic purposes; therefore, we could not influence the time of sampling. The earliest time point when we obtained CSF was day 14 after the onset of IVH, leaving us without information about the critically important first 2 weeks following IVH. We can speculate that in the first 2 weeks after IVH, we could have seen even higher amounts of Hb and its derivatives in the CSF. Another limitation of this work is that we have focused on only Hb and its derivatives, although it is very likely that the IVH-associated inflammatory response is much more complex. The major determinant of the inflammatory response and the outcome of IVH is the volume of bleeding, which likely correlates with the levels of Hb and its derivatives in the CSF. Despite these limitations, we believe that extracellular Hb and its derivatives contribute to the pathogenesis of IVH and pharmacological interventions targeting extracellular Hb, Hb oxidation, and heme could have a potential to limit the neuroinflammatory response following IVH.

## Data Availability Statement

All datasets generated for this study are included in the article/supplementary material.

## Ethics Statement

The procedures were approved by the Scientific and Research Ethics Committee of the University of Debrecen and the Ministry of Human Capacities under the registration number of 1770-5/2018/EÜIG. Parental consent forms were signed by the parents of the eight infants involved in this study.

## Author Contributions

JE: collection and assembly of data and participation in drafting the manuscript writing. AN, LN, and LB: patient selection, sample collection, and revision of the manuscript. AT, BNy, EB, and ZF: data collection and analysis. BNa, JK, AB, and GP: data analysis and interpretation, and revision of the manuscript. VJ: conception and design, data analysis and interpretation, and drafting the manuscript. All authors agreed to be accountable for all aspects of the work in ensuring that questions related to the accuracy or integrity of any part of the work are appropriately investigated and resolved. All authors approved the manuscript submission.

## Conflict of Interest

The authors declare that the research was conducted in the absence of any commercial or financial relationships that could be construed as a potential conflict of interest.
